# Large-Scale Molecular Dynamics Simulations Reveal New Insights Into the Phase Transition Mechanisms in MIL-53(Al)

**DOI:** 10.3389/fchem.2021.718920

**Published:** 2021-08-27

**Authors:** Sander Vandenhaute, Sven M. J. Rogge, Veronique Van Speybroeck

**Affiliations:** Center for Molecular Modeling (CMM), Ghent University, Ghent, Belgium

**Keywords:** soft porous crystals, phase transitions, transition mechanism, phase nucleation, phase propagation, molecular modeling, crystal size

## Abstract

Soft porous crystals have the ability to undergo large structural transformations upon exposure to external stimuli while maintaining their long-range structural order, and the size of the crystal plays an important role in this flexible behavior. Computational modeling has the potential to unravel mechanistic details of these phase transitions, provided that the models are representative for experimental crystal sizes and allow for spatially disordered phenomena to occur. Here, we take a major step forward and enable simulations of metal-organic frameworks containing more than a million atoms. This is achieved by exploiting the massive parallelism of state-of-the-art GPUs using the OpenMM software package, for which we developed a new pressure control algorithm that allows for fully anisotropic unit cell fluctuations. As a proof of concept, we study the transition mechanism in MIL-53(Al) under various external pressures. In the lower pressure regime, a layer-by-layer mechanism is observed, while at higher pressures, the transition is initiated at discrete nucleation points and temporarily induces various domains in both the open and closed pore phases. The presented workflow opens the possibility to deduce transition mechanism diagrams for soft porous crystals in terms of the crystal size and the strength of the external stimulus.

## 1 Introduction

Over the last decades, metal-organic frameworks (MOFs) have emerged as an extraordinary class of materials as a result of their unique building block concept from metal nodes and organic linkers, giving rise to an almost limitless number of materials (Chui et al., 1999; Li et al., 1999; Férey, 2001; Yaghi et al., 2003; Kitagawa et al., 2004). MOFs may display anomalous responses to external triggers and exhibit for example negative linear compressibility, negative thermal expansion, negative gas adsorption, or large-amplitude structural transformations while retaining their structural integrity (Coudert, 2015; Balestra et al., 2016; Bennett et al., 2017; Liu et al., 2018; Burtch et al., 2019; Coudert and Evans, 2019; Evans et al., 2019). Such stimuli-responsive behavior has been explored in various research fields including adsorption, separation, catalysis, and drug delivery (Horike et al., 2009; Li et al., 2009; Horcajada et al., 2012; Schneemann et al., 2014). More in particular, the terminology “soft porous crystals” (SPCs) was coined by Kitagawa and colleagues referring to those materials that exhibit bistable or even multistable behavior, possessing the ability to undergo large structural transformations upon exposure to external stimuli while maintaining their long-range structural order (Horike et al., 2009). Enormous experimental and computational research efforts have been undertaken to understand and ultimately predict or tune this functional behavior. However, most of our insights so far originate from thermodynamic considerations, while the mechanistic details of such large-amplitude phase transformations are yet to be resolved. For example, it is still unclear how the transformation nucleates and how this nucleation is affected by the presence of defects or the size of the crystal. Experimentally, a series of *in situ* experiments have established that the dynamic response of these materials is strongly affected by the presence of defects and the crystal size, whereby crystal downsizing was found to suppress their ability to morph (Sakata et al., 2013; Miura et al., 2017; Krause et al., 2018; Ehrling et al., 2019; Kundu et al., 2019; Wannapaiboon et al., 2019; Bon et al., 2020; Krause et al., 2020; Ehrling et al., 2021).

**FIGURE 1:**
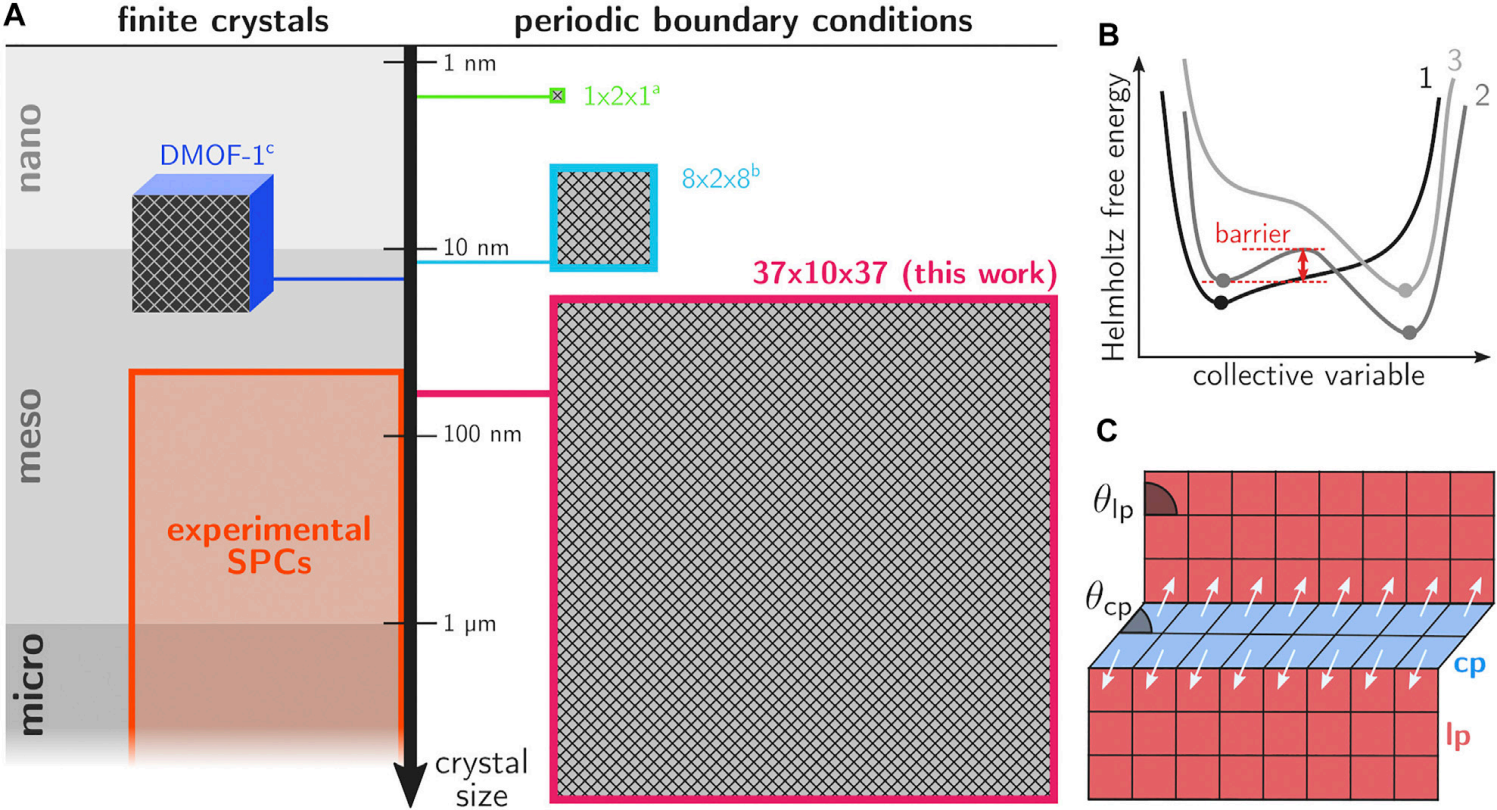
**(A)** Overview of the different length scales of computational models that were used in prior work on phase transitions in SPCs, together with the current supercell configuration and the range of experimental crystal sizes of MOFs. For MIL-53(Al), the 1 × 2 × 1 cell was employed in Vanduyfhuys et al. (2018) and the 8 × 2 × 8 cell in Rogge et al. (2019). The DMOF-1 crystallite is discussed in Keupp and Schmid (2019). **(B)** Conceptual Helmholtz free energy curves for conditions for which the material shows a single stable phase (curves 1 and 3) or two (meta)stable phases separated by an energy barrier (curve 2). **(C)** Schematic illustration of the layer-by-layer transition mechanism in MIL-53(Al).

Another major point of discussion regarding the transition mechanism concerns the degree to which the transition occurs in a collective, cooperative way. In many literature references, the hypothesis of collective behavior was assumed, where the entire framework transforms cooperatively (Schneemann et al., 2014; Wieme et al., 2018; Vanduyfhuys et al., 2018). Insights into the thermodynamics of the phase transformations have been obtained from atomistic simulations by constructing the Helmholtz free energy as function of the state variables governing the observed behavior (Rogge et al., 2015; Vanduyfhuys et al., 2018; Hobday and Kieslich, 2021). Figure 1B shows a series of conceptual free energy curves in terms of a collective variable that is able to uniquely distinguish between all (meta)stable or activated states. For many frameworks the unit cell volume proved to be an appropriate collective variable (Demuynck et al., 2018), however, this may not always be the case. For flexible linkers, more advanced procedures were necessary to find collective variables that allowed to describe the phase transformation. The study of such frameworks is beyond the scope of the current contribution. Herein, we will focus on MIL-53(Al), the prototypical flexible framework where transitions may be induced by temperature, pressure and gas adsorption (Demuynck et al., 2017, Demuynck et al., 2018). Experimentally determined structural parameters are presented in Supplementary Section S1. From the perspective of these Helmholtz free energy curves, collective behavior implies that any small barrier in the thermodynamic potential of a single unit cell would translate into a huge barrier for the entire system and the framework would only transition in case one of the minima in the thermodynamic potential would disappear (curves 1 and 3 in Figure 1B).

Nowadays, there are various indications that transitions in SPCs do not occur in a fully cooperative way. Early on, Triguero et al. proposed a theoretical layer-by-layer transition model for winerack frameworks like MIL-53(Al), where collective transformations between the large pore (lp) and closed pore (cp) phases only occur within a single layer at a time (Triguero et al., 2011). Their findings were based on a model that exploits the characteristic geometry of winerack frameworks, which are composed of rhombus-shaped cells in the directions perpendicular to the aluminum oxide chain. The shape of the rhombus cells can be characterized by the angle *θ*, which varies between 79° for the lp phase and 40° for the cp phase (see Figure 1C). The edge lengths do not change appreciably because the corresponding benzene-1,4-dicarboxylate (BDC) linkers do not allow for large variations in length. These specific geometrical features impose significant constraints on the possible deformation modes of the crystal if its crystallinity is to be preserved; as only those deformations are allowed which preserve the rhombus shape. With these assumptions, cells in a layer must deform coherently in order to preserve the lattice integrity. As such, it was concluded that the phase transformation occurs in an avalanche manner; once the lp-to-cp nucleation occurs at one point, the entire layer quickly transforms to the cp phase. Further lp-to-cp transformations were predicted to occur most likely in layers neighboring already transformed layers. More recent work has constructed energy versus volume curves for MIL-53(Al) using very accurate many-body dispersion calculations within the random-phase approximation (Wieme et al., 2018). Even at 0 K, a non-negligible barrier of about 8 kJ/mol was found between the lp and cp phase, indicating the existence of metastable lp crystals even at very low temperatures. This conclusion is corroborated by Mendt et al. who observed a fraction of lp material for MIL-53(Al) even at 9 K (Mendt et al., 2010).

Computational modeling can help to unravel such mechanistic details, provided that the employed models are realistic representations of experimentally observed MOFs, with dimensions comparable to real crystallites and with the explicit inclusion of the external surface and potential defects. Unfortunately, even with the massive amount of computing resources that is now available, such simulations are not yet feasible (Van Speybroeck et al., 2021). At present, the attainable length scales within the field of nanostructured materials are limited to a few nanometers and common molecular dynamics (MD) runs extend from the picoscale (for ab initio based methods) to the nanoscale range (for force field based methods). In the MOF field, the largest force field based simulations were performed on systems of about 10,000 atoms, with a length scale of tens of nanometers in one direction. Such spatial dimensions are still below experimental crystal sizes which may extend well into the micrometer range (see Figure 1A). As a consequence of these model limitations, some effects which are inherently related to (long-range) spatial disorder are not properly accounted for in current computational models (Van Speybroeck et al., 2021).

Recent work has attempted to simulate phase transformations in SPCs in a more realistic manner (see Figure 1A). Schmid et al. performed the first finite size simulations on nanocrystallites of DMOF-1(Zn) by setting up a crystallite model containing roughly 250,000 atoms, with explicit inclusion of the external surface (Keupp and Schmid, 2019). Such simulations abandon the periodic boundary conditions (PBCs) that are commonly employed in MOF computational modeling. A series of simulations were performed in a temperature ramp between 300 K and 500 K to observe the thermal opening, and constrained simulations were performed to observe the mechanical closing of the DMOF-1 system. They concluded that the transition nucleates near the external surface, where an interface between the cp and lp phase occurs which then travels dynamically through the lattice. The nucleation was found to be distinctively dependent on the surface-to-volume ratio. Such observations would not have been possible based on simulations with PBCs and small-sized unit cells, as spatial disorder is in that case not allowed and the external surface not taken into account. Simultaneously, Rogge et al. performed large scale force-field-based MD simulations on a series of MOFs, including MIL-53(Al), DMOF-1(Zn) and CoBDP using PBCs, with supercells that contained over 10,000 atoms (Rogge et al., 2019). The latter supercells were referred to as mesocells and their behavior was contrasted with nanocells having substantially smaller dimensions (see Figure 1A). Although the mesocells are still about an order of magnitude smaller than experimental crystal sizes, new physicochemical phenomena were observed. For the nanocell, the lp-to-cp transition occurs cooperatively and any form of spatial disorder is prevented by the enforced PBCs. On the other hand, when mesocells are exposed to pressures of 40 MPa at 300 K, part of the system undergoes an lp-to-cp transition whereas the rest temporarily remains in its original lp phase. Temporary interfacial defects were observed near the lp-cp phase boundary, which traverse the lattice until the transformation is complete. The phenomenon was also thermodynamically investigated by constructing the Helmholtz free energy profiles as a function of the volume, which strongly depend on the possibility of phase coexistence occurring in the larger mesocells. The latter phenomenon has not yet been confirmed experimentally, as this requires dedicated in situ cells where the temporal behavior of the material could be followed with very high spatiotemporal resolution.

From this discussion, it is clear that a full mechanistic understanding of the phase transformations in finite MOF crystals requires models that are substantially larger than the ones considered so far, as these would allow us to include spatial disorder at various length scales. Ultimately, simulations of finite-sized crystals containing millions of atoms and having sizes comparable to small experimental crystals would become feasible. In this work, we take an important step in this direction by exploiting the massively parallel architecture of state-of-the-art GPUs in order to simulate these phase transitions at unprecedented length scales. GPU acceleration is known to increase the attainable time and length scale of MD simulations by roughly two orders of magnitude as compared to a CPU, which is achieved by offloading the evaluation of interatomic forces and possibly the time integration onto the GPU. While most molecular mechanics engines nowadays provide some level of GPU acceleration, they are rarely compatible with the specialized force fields and thermodynamic ensembles that are required in common SPC simulation pipelines. In this work, we succeeded in porting the necessary force fields and sampling protocols to the highly extensible and GPU-oriented OpenMM software package (Eastman et al., 2017). In particular, this included the development and implementation of an extension of the existing pressure control algorithm towards fully anisotropic unit cell fluctuations. The proposed workflow enabled us to go far beyond currently accessible atomistic force field based MD simulations on MOFs. Herein, we perform simulations on models containing 1,040,440 atoms and a unit cell size of 54.9 × 6.6 × 45.1 nm^3^, thereby entering the range of small experimental crystals (Figure 1A). Visualization and analysis of the transition dynamics become increasingly challenging for such large systems. Herein, we present a dynamic two-dimensional lattice representation that provides a fundamentally new perspective of the framework dynamics. Based on the presented workflow, we are able to give more insight into operative transition mechanisms for different values of the external stimulus.

The remainder of the paper is organized as follows: Section 2 presents the methodological advances that were necessary to achieve this goal, with in particular the derivation and implementation of the proposed pressure control algorithm and the automated dynamic lattice representation. Section 3 then discusses the analysis of the observed transition mechanisms; the concluding discussion and perspectives for future work are provided in Section 4.

## 2 Methods

The phase transition mechanism is investigated using MD simulations on a massive scale, with framework models containing up to a million atoms and thousands of pores, and over timescales on the order of 10 ns. Such simulation sizes are unprecedented for SPCs, and require state-of-the-art GPUs in order to become computationally feasible. While GPU-accelerated MD has been around for over a decade, accurate computational models of the large-amplitude transition in winerack-type frameworks additionally require (i) an efficient implementation of a number of specialized force field interactions that are not commonly found elsewhere, and (ii) full flexibility of the simulation cell, as the box vector lengths and angles vary significantly during the transition (Rogge et al., 2018). In this work, we chose to employ the OpenMM software package because of its highly versatile and extensible API, and its flexibility in supporting custom interactions, thereby satisfying the first requirement. The second requirement—fully flexible unit cells—is more complicated because OpenMM does not natively support fully anisotropic unit cell fluctuations. Moreover, the implementation of existing anisotropic barostats in OpenMM would require an extensive rewrite of the entire codebase because they require the virial stress at every step in order to operate properly. To avoid this, we chose to extend an existing isotropic pressure control algorithm with fully anisotropic fluctuations, because it is relatively easy to implement on GPUs and is compatible with custom interaction potentials. The theoretical framework of the proposed method as well as a series of validation experiments are presented in Section 2.1.

The underlying physical mechanisms of the transition are exposed by visualizing trajectories using a dynamic two-dimensional lattice representation. This provides a fundamentally new perspective on the transition dynamics and the corresponding induced disorder. An overview of the automated and GPU-accelerated visualization procedure is given in Section 2.2.

### 2.1 Pressure Control

For systems that are subject to periodic boundary conditions, constant pressure MD is achieved by dynamically adjusting the unit cell parameters throughout the simulation in order to maintain equilibrium with an externally applied pressure. The vast majority of current pressure control algorithms [see e.g., Berendsen et al. (1984), MTK (Martyna et al., 1994), Langevin (Feller et al., 1995), and Parrinello-Rahman (Parrinello and Rahman, 1981; Nosé and Klein, 1983)] determine the appropriate change in unit cell parameters based on the instantaneous virial stress of the system, which is both expensive to compute and not always readily available (e.g., in polarizable force fields). To avoid the virial computation, an alternative algorithm has been developed which instead performs Monte Carlo trial moves in the unit cell volume in order to sample the desired isothermal-isobaric phase space distribution (Chow and Ferguson, 1995; Åqvist et al., 2004). Here, we extend this Monte Carlo pressure control algorithm towards fully anisotropic cell fluctuations, as these are critical for the description of phase transformations in (soft) condensed matter and SPCs in particular (Rogge et al., 2018).

#### 2.1.1 Derivation

The Monte Carlo pressure control algorithm works by performing Metropolis sampling in the unit cell degrees of freedom during a MD simulation, with trial moves being performed every 10 to 100 steps. Whereas previous work only considered isotropic moves, i.e., trial moves which attempt to scale the unit cell isotropically while leaving its shape unaltered, we here present a more general approach that considers trial moves in all unit cell degrees of freedom. We begin the derivation by considering a molecular system that is periodic in all three dimensions, with a triclinic unit cell that contains *N* atoms. A microstate of this system is determined by the cartesian coordinates of its *N* particles $r=\left(r_{1},r_{2},\ldots ,r_{N}\right)$ and, in addition, three linearly independent box vectors ***a***, ***b***, and ***c*** which determine the periodicity of the system. These box vectors can be arranged along the rows of a 3 × 3 cell matrix ***h***, in which case the unit cell volume is given by $V=\det\left(h\right)$. The configurational partition function in the isothermal-isobaric ensemble for this system is then defined as (Martyna et al., 1994; Tuckerman, 2010):

$$\Delta \left(N,P,T\right)=C\iint e^{-\beta U\left(r\right)}e^{-\beta PV}\det{\left(h\right)}^{-2}\text{d}h\text{d}r$$ (1)

whereby *β* = (*kT*)^−1^ represents the inverse temperature, *P* the externally applied pressure, and *C* a constant that is otherwise irrelevant. The differentials d***h*** and d***r*** that appear in Eq. 1 represent integrations over the nine components of the matrix ***h*** and the 3*N* components of the coordinates ***r***, respectively. Most MD engines require cell matrices to be in lower triangular form due to efficiency considerations, and it is therefore necessary to first rewrite Eq. 1 in terms of lower triangular cell matrices ***h***_△_:

$$h_{△}=\left[\begin{array}{ccc} a_{x} & 0 & 0\\ b_{x} & b_{y} & 0\\ c_{x} & c_{y} & c_{z} \end{array}\right]$$ (2)

This may be achieved by eliminating global rotations of the coordinate system. As explained in Supplementary Section S2, this enables us to transform the original nine-dimensional integration over ***h*** into a six-dimensional integration over ***h***_△_:

$$\Delta \left(N,P,T\right)=C\iint e^{-\beta U\left(r\right)}e^{-\beta PV}{\left(b_{y}c_{z}^{2}\right)}^{-1}\text{d}h_{△}\text{d}r$$ (3)

Eq. 3 represents the desired phase space distribution that we wish to approximate. To derive a specific expression for the acceptance probability of a given trial move in a Metropolis random walk, we first need to remove the ***h***_△_-dependence in the integration limits of the particle coordinates; a similar procedure is necessary when deriving acceptance probabilities in regular isothermal-isobaric Monte Carlo (Tuckerman, 2010). This may be achieved by defining normalized particle coordinates ***s***_*i*_ for each particle *i* (in components):

$$\begin{array}{ll} r_{i,x} & =a_{x}s_{i,x} \end{array}$$ (4)

$$\begin{array}{ll} r_{i,y} & =b_{y}s_{i,y} \end{array}$$ (5)

$$\begin{array}{ll} r_{i,z} & =c_{z}s_{i,z} \end{array}$$ (6)

and after performing the substitution in Eq. 3:

$$\Delta \left(N,P,T\right)=C\iint e^{-\beta U\left(s,h_{△}\right)}e^{-\beta PV}{\left(b_{y}c_{z}^{2}\right)}^{-1}V^{N}\text{d}h_{△}\text{d}s$$ (7)

whereby both integrations are now performed over fixed domains. Eq. 7 now allows us to use Metropolis sampling to generate a Markov chain of unit cell matrices that exhibits the correct phase space distribution (Metropolis et al., 1953). A trial move ***h***_△*o*_ → ***h***_△*n*_ is generated by sampling random displacements from a uniform distribution in all six components of the unit cell. The move is then accepted with a probability derived from Eq. 7:

$$\mathrm{Pr}\left[h_{△o}\to h_{△n}\right]=\min\left[1,\exp\left(\overset{3}{\underset{i=0}{\sum }}c_{i}\right)\right]$$ (8)

with

$$\begin{array}{ll} c_{0} & =-\beta \Delta U \end{array}$$ (9)

$$\begin{array}{ll} c_{1} & =-\beta P\Delta V \end{array}$$ (10)

$$\begin{array}{ll} c_{2} & =N\log\left(\frac{V_{n}}{V_{o}}\right) \end{array}$$ (11)

$$\begin{array}{ll} c_{3} & =\log\left(\frac{{\left(b_{y}c_{z}^{2}\right)}_{n}^{-1}}{{\left(b_{y}c_{z}^{2}\right)}_{o}^{-1}}\right) \end{array}$$ (12)

and where Δ*U*, Δ*V* denote the differences in potential energy and volume between the new unit cell ***h***_△*n*_ and the old unit cell ***h***_△*o*_. The amplitude of the displacements is dynamically adjusted such that, on average, about 50% of the trial moves are accepted.

#### 2.1.2 Validation

The isothermal-isobaric ensemble is characterized by a phase space distribution in accordance with Eq. 1, and the here proposed pressure control algorithm should succeed in generating phase space trajectories that are distributed accordingly. To critically verify that this is indeed the case, we performed a number of validation experiments on strongly anisotropic systems.

First, we considered a simple harmonic crystal in which only short-range covalent interactions are present. The force constants and geometry of the system were deliberately chosen as to induce a large degree of anisotropy, with fluctuations in unit cell parameters that are sufficiently large. MD trajectories obtained at different pressures were then evaluated based on the convergence of the average stress tensor and by following the rigorous ensemble validation protocol outlined in Shirts (2013) and Merz and Shirts (2018). As reported in Supplementary Section S3, all trajectories passed the stress tensor convergence and ensemble validation tests.

**FIGURE 2:**
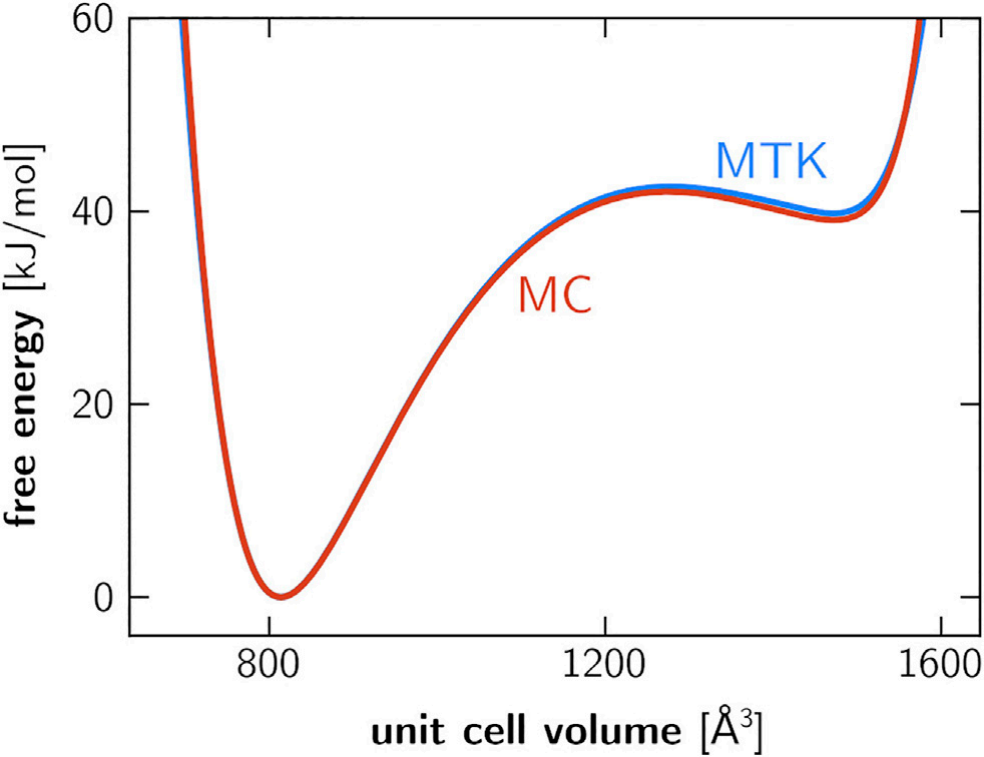
Helmholtz free energy of a 1 × 2 × 1 unit cell of MIL-53(Al), computed using umbrella sampling (Supplementary Section S3). The blue curve is obtained based on simulations that were performed using the MTK barostat, whereas the red curve was obtained using the proposed Monte Carlo barostat. The discrepancy in relative stability between the lp and cp phases is around 0.9 kJ/mol. While simulations were performed on the 1 × 2 × 1 cell, the unit cell volume axis employed here refers to the 1 × 1 × 1 cell.

A second validation experiment was performed specifically on the MIL-53(Al) framework. Previous work has established the importance of anisotropic fluctuations in the relative stability of the lp and cp phases (Rogge et al., 2018), and it is imperative that the Monte Carlo based pressure control algorithm yields the same relative stabilities as virial-based barostats (e.g., MTK or Langevin). To demonstrate their equivalence, we computed the Helmholtz free energy as a function of the unit cell volume using umbrella sampling, employing either the proposed Monte Carlo barostat or the existing MTK barostat (Supplementary Section S4). As observed in Figure 2, both approaches are equivalent as the predicted relative differences in cp-lp stability deviate by less than two percent.

Both validation experiments provide strong evidence for the correctness of the derivation and implementation of the proposed pressure control algorithm. It is worth mentioning that throughout the derivation, no modifications to the equations of motion of individual particles were necessary. This suggests that the effect of Monte Carlo pressure control on the overall dynamics of the system is expected to be negligible, which has indeed been confirmed for the isotropic variant based on the invariance of diffusion coefficients (Chow and Ferguson, 1995). Lastly, an important advantage over virial-based approaches is that the proposed method works equally well for simulations in which forces and energies are computed in single precision, whereas virial-based barostats may in that case experience overall drifts in e.g. the average density (as discussed in Harger and Ren (2019)). This is particularly important for GPU acceleration, as it is well known that the floating point performance of GPUs in single precision is much higher as compared to double precision (Le Grand et al., 2013).

### 2.2 Visualization

**FIGURE 3:**
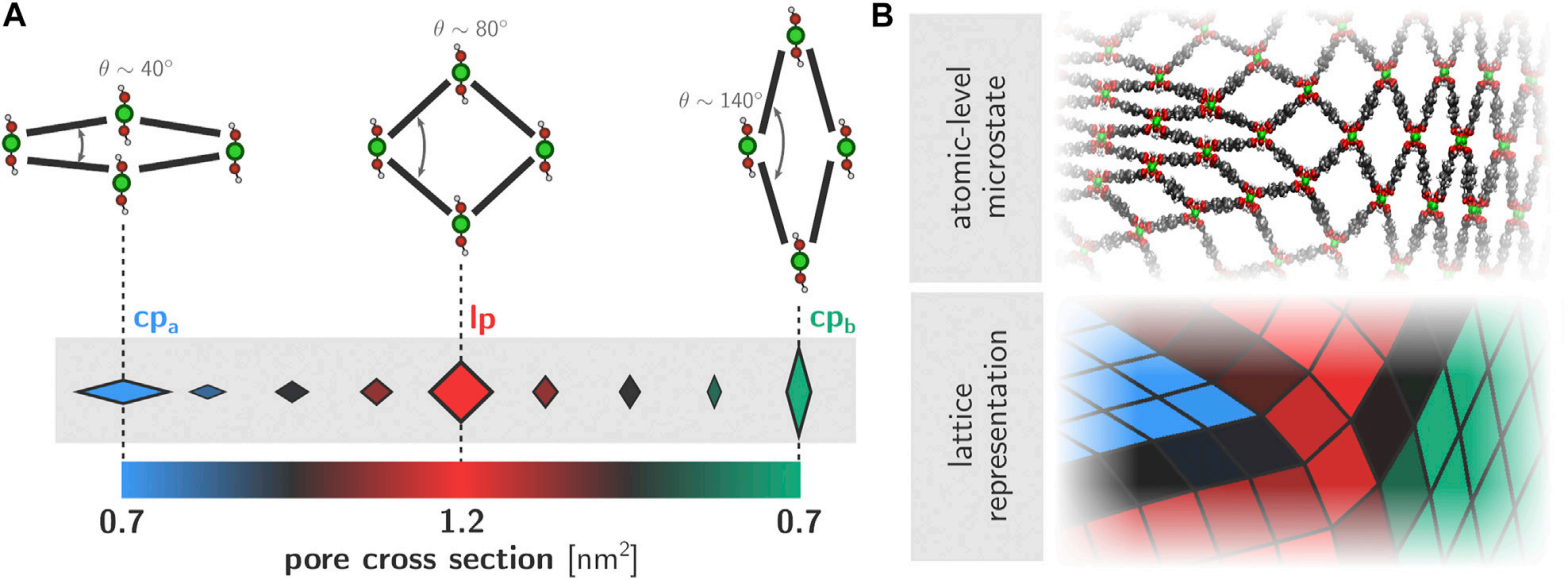
**(A)** Quadrilateral representation of individual one-dimensional channels within the wine rack topology of MIL-53(Al). The color of each quadrilateral is determined based on the instantaneous cross-sectional area of the corresponding channel **(B)** Example configuration of the framework and corresponding two-dimensional lattice representation.

A physically accurate and visually clear representation of the obtained trajectories is vital in order to understand the physical mechanisms that govern the phase transition behavior. Previous work has established how both the transition itself as well as the various forms of induced disorder are essentially two-dimensional phenomena that are translationally invariant along the direction of the aluminum chains (Rogge et al., 2019). As such, we chose to visualize the framework dynamics using a two-dimensional representation of the lattice, where the aluminum chains are represented by vertices and the adjoining organic ligands by edges (Figure 3). The position of the vertices is obtained by projecting the corresponding aluminum chain onto the cross-sectional plane. This two-dimensional representation of the framework structure is then constructed for each snapshot in a given trajectory. In order to further highlight the state of each of the pores in the system, we color each quadrilateral based on its instantaneous cross-sectional area. More specifically, the lp and both of the cp phases are filled using full colors (respectively red, blue, and green). For pores that are transitioning between phases and therefore contain cross-sectional areas that are between the pure lp and cp values, we used different shades of gray as indicated in the colorbar in Figure 3A. As such, the framework dynamics and local phase behavior are visualized and exposed in a tangible manner.

### 2.3 Computational Details

#### 2.3.1 Force Fields

All simulations on the MIL-53(Al) framework were performed using an ab initio derived force field. The covalent interactions were obtained based on quantum mechanical input data using the QuickFF protocol (Vanduyfhuys et al., 2015). The electrostatic interactions were included by considering Gaussian charge distributions around each atom. The magnitude of the charge distributions was obtained using the Minimal Basis Iterative Stockholder (MBIS) scheme (Verstraelen et al., 2016), and the radius of the distributions were obtained using the scheme by Chen and Martínez et al (2007). Dispersion interactions were modelled using MM3-type interactions (Allinger et al., 1989). The force field has been validated extensively in previous work (Vanduyfhuys et al., 2018). The OpenMM input files for the force field were prepared using the OpenYAFF conversion tool (Vandenhaute, 2021b).

#### 2.3.2 Molecular Dynamics Simulations

Large-scale MD simulations on the transition mechanism of MIL-53(Al) were performed using OpenMM 7.5.0 (Eastman et al., 2017), supplemented with the implementation of the new barostat (available online (Vandenhaute, 2021a)). The simulations were performed using a leapfrog Langevin integrator with a friction coefficient of 0.1 ps^−1^ (Zhang et al., 2019) and a timestep of 0.5 fs. Monte Carlo trial moves in the unit cell degrees of freedom were performed every five steps. With this trial move frequency, it takes roughly 10 ns of simulation time for a full lp–cp transition. Particle positions and unit cell vectors were sampled every 5 ps. Dispersion interactions were smoothly truncated at 11 Å, and supplemented with analytical tail corrections. Electrostatic interactions were evaluated using the particle mesh Ewald (PME) method, with a splitting parameter *α* of 0.32 Å^−1^ and a reciprocal space cutoff of 0.35 Å^−1^. All simulations were performed on a single NVIDIA V100 GPU with 32 GB of memory, achieving a simulation speed of about 0.5 ns/day in mixed precision mode, in which case forces and energies are computed in single precision and the time integration is performed in double precision, thereby achieving an optimal tradeoff between accuracy and computational efficiency (Eastman et al., 2017). We note that no additional free energy calculations were performed beyond the validation in Figure 2, as these are not required to understand the different transition mechanisms. Furthermore, the unit cell volume is no longer a suitable collective variable for systems of this size given that the statistical fluctuations in *V* are almost entirely absent due to the very large number of particles.

## 3 Results

We investigated the transition mechanism based on large-scale MD simulations at constant temperature and pressure. In order to eliminate PBC artefacts as much as possible and ensure that phase separation and/or coexistence is maximally allowed by the model, we performed the simulations on a 37 × 10 × 37 unit cell containing 1,040,440 atoms and just over 2,500 individual pores. Such unit cell sizes are unprecedented in computational research on MOFs and SPCs in particular, and represent a significant step forward with respect to the state-of-the-art (as visualized in Figure 1A). To identify the pertinent features in the framework dynamics, we simulated the lp-to-cp transition for this system at different thermodynamic conditions. An overview of all simulations performed with their specific control variables is given in Supplementary Table S2. Specifically, we considered three different temperatures (200, 300 and 500 K) and two different pressures for each temperature (100 and 300 MPa). While both pressures are much higher than the experimentally observed transition pressure for this material—which is estimated at about 13–18 MPa at room temperature (Yot et al., 2014)—these pressures ensured that the phase transition proceeds sufficiently fast as to make the simulations computationally feasible on single-GPU systems. Alternatively, enhanced sampling techniques may be used to speed up the transition, for example by biasing the dynamics of the framework along the *θ* angle indicated in Figure 3. As this is a first case study of the large-scale dynamics of winerack-type frameworks, we chose not to pursue this direction and consider regular unbiased dynamics at elevated transition pressures instead. Nevertheless, the transition times of the employed 37 × 10 × 37 system are observed to be roughly three orders of magnitude larger as compared to a fully cooperative transition in a 1×2×1 cell (∼10 ns versus ∼10 ps).

**FIGURE 4:**
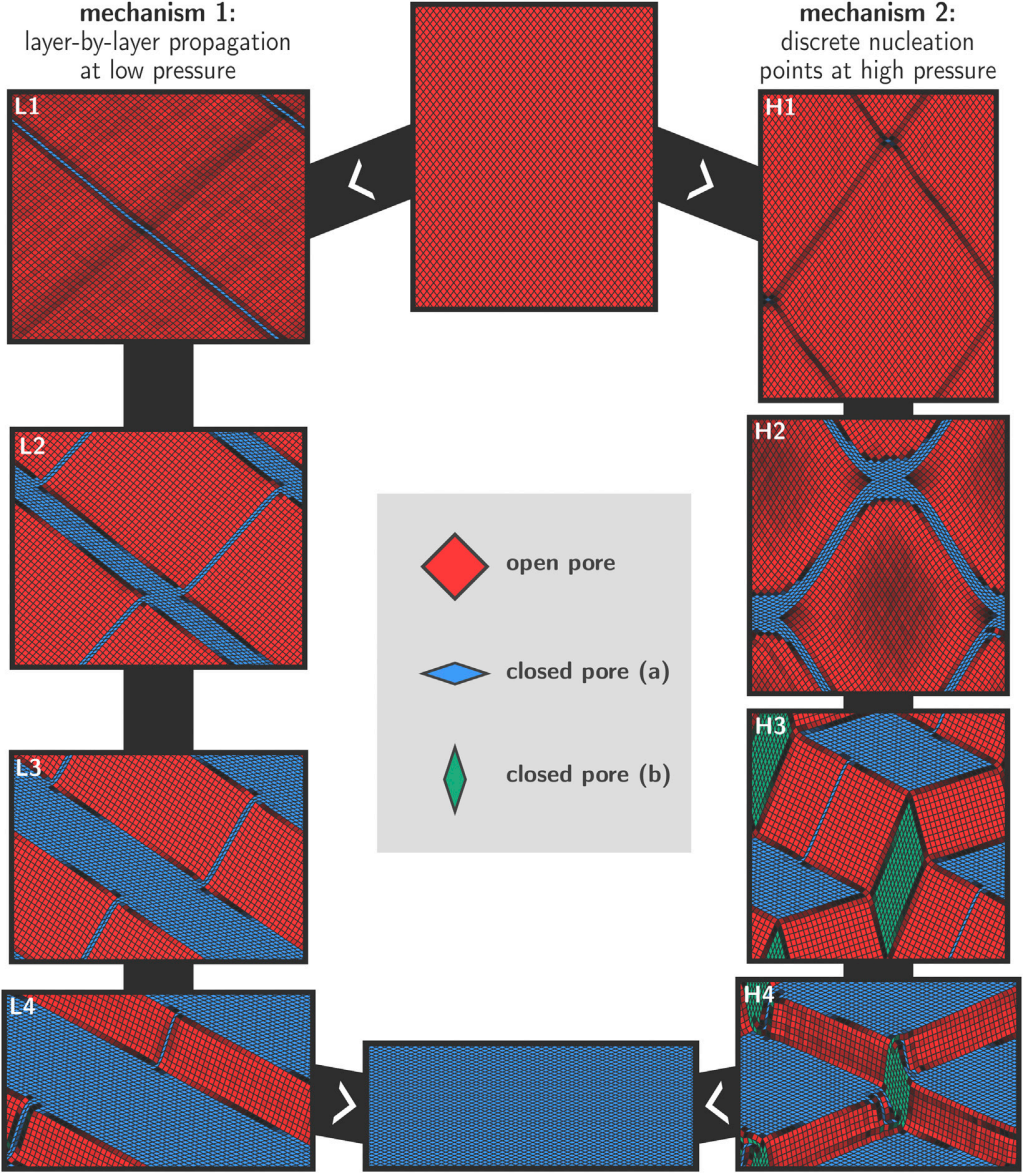
Visualization of the two observed transition mechanisms for the lp–cp transformation in MIL-53(Al), as explained in Section 2.2. For lower pressures, a layer-by-layer mechanism is preferred in which the rhombus shape of the pores is approximately preserved throughout the transition **(left)**. For higher pressures, we observe discrete nucleation points and the formation of cp and lp domains **(right)**.

Figure 4 shows snapshots of the transition mechanisms at 300 K in the low- and high-pressure regimes, as obtained from simulations at 100 MPa (left) and 300 MPa (right). The final aim is to unravel more details about the transition mechanisms at various conditions. Before discussing the results in detail, we first provide some general remarks on the transition based on topological considerations. Considering the winerack-type structure of the framework and the very limited intrinsic flexibility of the BDC linkers in between aluminum chains, physically feasible transition mechanisms need to preserve the approximately rhombus shape for most of the pores in the system because the energy required to strongly deform the cells from their rhombus shape is expected to be very high. In addition, interatomic forces were modelled using a classical force field and as such we implicitly enforce strict covalent bond integrity throughout the entire transition. Deviations from the rhombus shape might eventually be possible provided that the mechanical energy supplied to the system is sufficiently high. Our simulations show that essentially two different transition mechanisms are active depending on the magnitude of the applied external pressure. At lower pressures, a layer-by-layer transition mechanism is observed whereas at higher pressures discrete nucleation points emerge within the lattice, which ultimately give rise to domain formation during the transformation. These results are schematically shown in Figure 4, where the left column shows the snapshots from the 100 MPa transition, and the right column shows the snapshots from the 300 MPa transition. While both transitions were recorded at 300 K, we observed entirely similar behavior at 200 K and at 500 K.

### 3.1 Layer-By-Layer Transition at Low Pressures

For all transitions with relatively low values of the external stimulus (100 MPa); we observe a diagonal layer-by-layer mechanism in agreement with the conceptual model proposed in Triguero et al. (2011). This mechanism is initiated in one layer in which all cells switch cooperatively from the lp to the cp phase (Figure 4, panel L1). The formation of the initial cp layer within an lp bulk phase creates two-dimensional phase boundaries that extend diagonally across the material. It may be noted that a second gray-shaded diagonal appears. However, in this pressure regime, this region does not increase substantially during the simulation.

**FIGURE 5:**
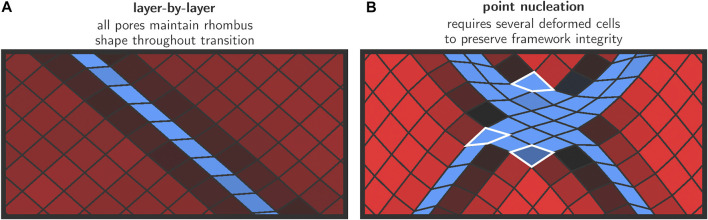
Snapshots for the transition mechanisms in the initial growth phase. For the layer-by-layer mechanism **(A)**, the rhombus shape is preserved throughout the transition. In the case of discrete nucleation points **(B)**, large deformations are necessary to preserve the framework integrity—such cells are emphasized with a white border.

To obtain more insight into the deformation mode of the various cells in and around the layer where the transformation was initiated, an enlarged representation of this area in the lattice is shown in Figure 5A. It can be seen that all unit cells maintain their rhombus-like shape and that only the angle *θ* changes substantially from ca. 78° to ca. 42°. The linkers approximately maintain their length. This is in line with our earlier hypothesis where we anticipated that only small deformations from a rhombus shape are energetically feasible at low pressures.

Once a cp layer has been formed, the transition proceeds gradually in the direction normal to the nucleation layer, whereby lp layers at the lp/cp phase boundary collapse and are appended by the growing cp region. This effectively leads to a propagation of the lp/cp phase boundary in the direction normal to the nucleation layer (Figure 4, panels L2–L4). This process continues until all layers have transformed to the cp phase, at which point the transition is complete and a minimum in free energy is encountered. Such a layer-by-layer mechanism does not require individual pores to deviate from their rhombus shape, and their equilateral form is preserved at all times during the transition. The only internal coordinate that varies throughout the entire process is the angle *θ*.

As already indicated earlier, it is clear from the visualization in panels L1 to L4 of Figure 4 that a second diagonal having the cp phase is formed parallel to the growth direction and roughly orthogonal to the existing growing cp region. This second cp region does not propagate further throughout the material, and does not otherwise mediate the transition in a significant way. We therefore hypothesize that it is a result of the rather large pressures that had to be applied in order to make the transition computationally feasible. The applied pressure of 100 MPa in the left panels of Figure 4 is still significantly higher than the lowest possible transition pressure which was previously established at around 30 MPa (Vanduyfhuys et al., 2018), and the appearance of a second cp diagonal therefore appears to be a way for the framework to release the excessive strain within the lattice.

### 3.2 Transition via Discrete Nucleation Points at High Pressures

At significantly higher pressures, an alternative transition pathway was observed where the transition is initiated at various nucleation points and where also various domains are formed in the crystal. The snapshots resulting from a MD simulation at 300 K and 300 MPa are shown in panels H1 to H4 in Figure 4. Similar observations were made for simulations performed at pressures above 300 MPa.

Early on in the simulation (panel H1), discrete nucleation points are formed throughout the framework. Immediately after their formation, these nucleation points become connected along various diagonals (panel H2). To obtain more insight into the deformation modes of the individual cells in this high-pressure regime, Figure 5B shows an enlarged representation of the deformed region around a nucleation point, in which we observe how some cells are strongly deformed from their original rhombus-like shape. This is possible because the energy required to induce such large deformations is now mechanically supplied to the system due to the high external pressure. This is in stark contrast with the layer-by-layer mixed phase configurations which were topologically allowed and did not require this type of deformations.

Also visible in panel H2 are the gray-shaded areas in between the interconnected nuclei, indicating that pores in those regions are significantly smaller than their full lp counterparts. We may regard these regions as being squeezed by nuclei on either sides due to the large structural difference between the cp and lp phase. At this stage, continued growth of the cp regions further increases the strain, until the point where domain formation occurs in which alternate cp (both with acute and obtuse *θ* angles) and lp domains are present (panel H3). As such, all three possible phases of the framework are found to coexist temporarily during this phase of the transition. The blue cp regions continue to grow at 300 K and 300 MPa, at the expense of the lp and alternative cp domains, which ultimately disappear at which point the transition is complete.

In spite of the enormous size of the mesocell, artefacts of the employed PBCs are still visible in the snapshots in Figure 4. For example, in panel H3, the structure and geometry of the different domains is necessarily organized such that the required periodicity is satisfied. Clearly, a full understanding of the transition dynamics requires computational models in which the implicit assumption of exact long-range order—as is the case when using PBCs—is completely eliminated, for example by using finite crystallites. In addition, we note that the dynamics of the system is not purely hamiltonian. Specifically, the temperature of the system was controlled using a Langevin thermostat, which implies that the equations of motion are modified with a stochastic contribution, the magnitude of which is determined by the friction coefficient (see Section 2.3). In addition to the thermostat, the dynamics of the system are also perturbed by the barostat, which performs small but frequent changes to the unit cell parameters. While we do not expect either the temperature or pressure control to induce drastic changes in the transition dynamics, they do prohibit a thorough analysis of the kinetics of the transition, as e.g., the transition time is dependent on the frequency of barostat trial moves.

## 4 Conclusion and Perspectives

**FIGURE 6:**
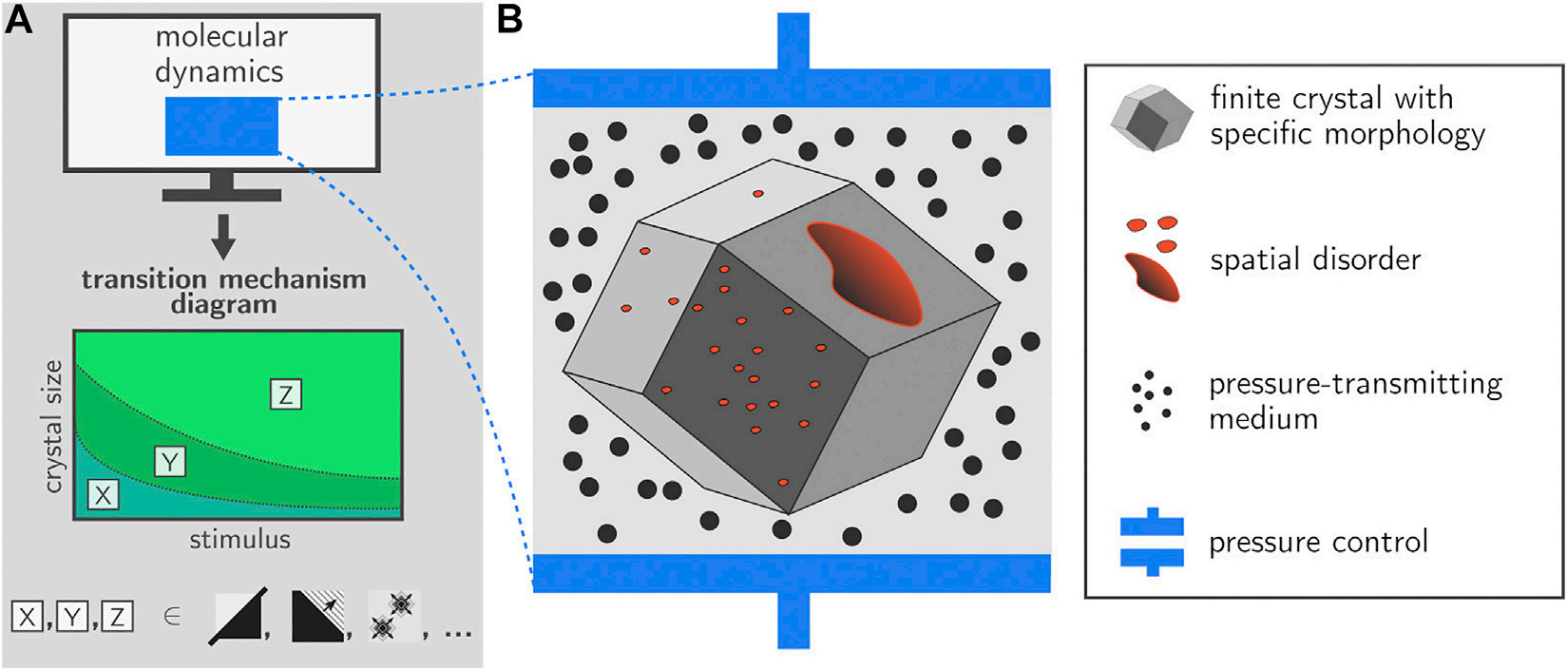
Future research on transition dynamics within framework materials should take into account the finite crystal size, various forms of spatial disorder, and the external surface. It should become possible to provide transition mechanism diagrams which illustrate which transition mechanisms are preferred as a function of control variables such as crystal size and external stimulus (e.g., fully cooperative, layer-by-layer, discrete nucleation points).

The simulations performed here constitute an important step forward towards a full mechanistic understanding of various phase transformations in realistic MOF particles. The implementation of the proposed pressure control algorithm in the GPU-accelerated OpenMM library as presented here allows to include fully anisotropic unit cell fluctuations in large-scale simulations containing millions of atoms. Its area of application is not limited to the breathing transition in winerack frameworks but is very general, and also includes e.g., crystalline-to-amorphous transitions. As such, we have taken a major leap forward in terms of the size of the systems that can be simulated in the field of MOFs. Based on the current simulations, we demonstrate that the transition mechanism may be critically dependent on various elements such as the size of the crystal but also the strength of the external stimulus, which is in this case the external pressure. For future simulations, it would be interesting to extend the protocol towards other stimuli such as temperature but eventually also guest molecules, and unravel whether similar transition mechanisms are observed. Such simulations might enable to construct a transition mechanism diagram that indicates the expected transition mechanism as a function of various control variables. Such a hypothetical diagram is illustrated in Figure 6A. The icons X,Y,Z indicate various mechanisms such as collective behavior, layer-by-layer behavior, or transitions starting from discrete nucleation points. Based on the simulations performed in this paper and literature data, we are already able to fix a number of points on the transition mechanism diagram in terms of the external pressure. However, more simulations are necessary to provide the complete transition mechanism diagram.

Despite the new insights obtained in this work, further methodological steps are necessary to enable the simulation of systems that are truly representative of realistic MOF particles, i.e., with crystal sizes similar to experimentally observed crystals, and with explicit inclusion of defects and the crystal surface. In what follows, we give some reflections on future perspectives in this direction.

First of all, the current approach will have to be extended towards finite nanocrystals with morphologies that are representative of experimentally observed crystallites and with length scales going beyond 50 nm. Subsequent embedding of this crystallite in a medium will then allow to apply an external pressure to the crystal in a very natural manner (Figure 6). Experimentally, both mercury as well as silicone oil have been used as effective pressure transducers in order to detect pressure-induced structural transitions in several winerack frameworks (Beurroies et al., 2010; Yot et al., 2012; Yot et al., 2014, 2016; Ramaswamy et al., 2017; Henke et al., 2018; Krause et al., 2018; Wahiduzzaman et al., 2018; Wieme et al., 2019; Yot et al., 2019) and other flexible frameworks such as ZIF-4 or DUT-8 (Kavoosi et al., 2017; Krylov et al., 2020); the simulation setup as described in Figure 6B would mirror such experiments.

Second, the current simulations were performed using an all-atom classical force field which does not allow any bond breakage to occur. It is not excluded that during a phase transition, bonds may temporarily break at specific locations within the framework as it is well known that linkers in MOFs may have a labile nature especially when being exposed to guest particles that allow to temporarily stabilize detached linkers (Hajek et al., 2018; Caratelli et al., 2019). To account for such effects, it is necessary to employ reactive force fields or more complex machine learning potentials (MLPs) that are trained based on quantum mechanical calculations in order to capture such effects. However, the systematic construction of MLPs for the complex systems under study here is highly nontrivial. To the best of our knowledge, only one MLP has so far been constructed for MOFs, by the group of Behler on MOF-5 (Eckhoff and Behler, 2019).

Finally, while current simulations allowed to deduce qualitatively new mechanistic details on the phase transition in MIL-53(Al), a next step would additionally aim to determine thermodynamic and kinetic properties associated with the transition, including its nucleation and growth. From a thermodynamic point of view, we could resort to the construction of Helmholtz free energy diagrams in terms of an appropriate collective variable. As noted in Section 2.3, the unit cell volume can no longer be regarded as an appropriate collective variable due to the absence of significant statistical fluctuations, and other variables will have to be considered such as the opening angle *θ*. The determination of kinetic properties, such as propagation rate constants for the phase boundaries, is highly challenging for these systems and may require specialized sampling protocols.

Previous considerations clearly illustrate the complexity associated with a full understanding of phase transformations in SPCs. The problem at hand is a prototypical example of a spatiotemporal process, where the dynamics of the MOF lattice is affected by spatial heterogeneities at various length and time scales (Van Speybroeck et al., 2021). A full understanding of the spatiotemporal response of MOFs will require a close partnership between the modeling and experimental community, whereby dedicated experimental *in situ* methods are necessary to track intermediate metastable states during their dynamic response towards external stimuli, and where theoreticians will have to explore new modeling avenues to tackle processes at various length and time scales.

## Data Availability

The raw data supporting the conclusions of this article will be made available by the authors, without undue reservation.
