# Supplementary material for: Large-Scale Molecular Dynamics Simulations Reveal New Insights Into the Phase Transition Mechanisms in MIL-53(Al)
Source: Front Chem. 2021 Aug 27;9:718920. doi: 10.3389/fchem.2021.718920 (PMC8429608; doi:10.3389/fchem.2021.718920)
Supplement: Supplementary file 1 [file DataSheet1.PDF]

Supplementary Material

1 EXPERIMENTAL PROPERTIES OF MIL-53(AL)

Table S1 gives an overview of the experimentally determined structural parameters of MIl-53(Al), as reported by Yot et al. (2014).

	**large pore**	**closed pore**
space group	*Imcm*	*C2/c*
a [Å]	16.7322(1)	19.633(1)
b [Å]	16.7322(7)	7.160(1)
c [Å]	6.6295(2)	6.559(1)
β [deg]	–	104.70(1)
volume [Å^3]	1423.8(1)	897.2(6)

Table S1. Experimental properties on the structure of MIL-53(Al) (Yot et al., 2014).

2 INTEGRATION OVER LOWER TRIANGULAR CELL MATRICES

This section demonstrates how we may write the isothermal-isobaric partition function into an integration over the six degrees of freedom of a lower triangular cell instead of the nine degrees of freedom for an arbitrarily oriented cell. To achieve this, we first need to define the transformation between the initial cell matrix $\boldsymbol{h}$ and the lower triangular cell matrix $\boldsymbol{h}_\triangle$. This transformation is conveniently obtained by computing the QR decomposition of the transpose of $\boldsymbol{h}$:

$$QR(\boldsymbol{h}^T) = \boldsymbol{q}\boldsymbol{h}_\triangle^T \tag{S1}$$

and with $\boldsymbol{q}$ the orthonormal rotation matrix. We then obtain:

$$\boldsymbol{h}_\triangle = \boldsymbol{h}\boldsymbol{q} \tag{S2}$$

$$= \begin{bmatrix} a_x & 0 & 0 \\ b_x & b_y & 0 \\ c_x & c_y & c_z \end{bmatrix} \tag{S3}$$

and for the volume of the unit cell:

$$V = \det(\boldsymbol{h}) \tag{S4}$$

$$= \det\left(\boldsymbol{h}_\triangle\right) \tag{S5}$$

$$= a_x b_y c_z \tag{S6}$$

Equation S3 defines the change of variables that must be performed in the integral of the partition function (repeated here for convenience):

$$\Delta(N, P, T) = C \int\int e^{-\beta U(\boldsymbol{r})} e^{-\beta PV} \det(\boldsymbol{h})^{-2} \, \mathrm{d}\boldsymbol{h}\mathrm{d}\boldsymbol{r} \tag{S7}$$

with C an irrelevant constant, N the number of particles in the unit cell, P the hydrostatic pressure, T the temperature, β the inverse temperature, and finally $U(\boldsymbol{r})$ the potential energy of the system as a function of the cartesian coordinates of all atoms (which contains an implicit dependence on the cell matrix). It is important to keep in mind that while equation S3 represents a simple rotation of the coordinate axes, the rotation matrix $\boldsymbol{q}$ is itself not a constant and instead depends on $\boldsymbol{h}$ (the integration variable), and this complicates the transformation of the differentials. The Jacobian of the change of variables in equation S3 has been computed elsewhere (see e.g. Edelman and Rao (2005), equation 3.6) and was found to be:

$$(\mathrm{d}\boldsymbol{h}) = a_x^2 b_y \left(\mathrm{d}\boldsymbol{h}_\triangle\right) \left(\boldsymbol{q}^T \mathrm{d}\boldsymbol{q}\right) \tag{S8}$$

Based on the obtained Jacobian, we may now perform the substitution suggested in equation S3:

$$\Delta(N, P, T) = C \int\int e^{-\beta U(\boldsymbol{r})} e^{-\beta PV} \det(\boldsymbol{h})^{-2} \,\mathrm{d}\boldsymbol{h}\mathrm{d}\boldsymbol{r} \tag{S9}$$

$$= C \int\int e^{-\beta U(\boldsymbol{r})} e^{-\beta PV} \det\left(\boldsymbol{h}_{\triangle}\right)^{-2} a_x^2 b_y \left(\mathrm{d}\boldsymbol{h}_{\triangle}\right) \left(\boldsymbol{q}^T \mathrm{d}\boldsymbol{q}\right) \mathrm{d}\boldsymbol{r} \tag{S10}$$

$$= C' \int\int e^{-\beta U(\boldsymbol{r})} e^{-\beta PV} \det\left(\boldsymbol{h}_{\triangle}\right)^{-2} a_x^2 b_y \left(\mathrm{d}\boldsymbol{h}_{\triangle}\right) \mathrm{d}\boldsymbol{r} \tag{S11}$$

$$= C' \int\int e^{-\beta U(\boldsymbol{r})} e^{-\beta PV} \left(a_x b_y c_z\right)^{-2} a_x^2 b_y \left(\mathrm{d}\boldsymbol{h}_{\triangle}\right) \mathrm{d}\boldsymbol{r} \tag{S12}$$

$$= C' \int\int e^{-\beta U(\boldsymbol{r})} e^{-\beta PV} (b_y c_z^2)^{-1} \left(\mathrm{d}\boldsymbol{h}_{\triangle}\right) \mathrm{d}\boldsymbol{r} \tag{S13}$$

whereby C' is introduced to absorb the constant integration over the rotation matrix $\boldsymbol{q}$. Equation S13 is identical to the result stated in equation 3 in the main text.

3 VALIDATION USING THE ANISOTROPIC CRYSTAL

The proposed pressure control algorithm is critically validated using a fictitious crystal that is designed to exhibit strong anisotropy. The triclinic unit cell of the crystal is described by the following cell matrix (in angstrom):

$$\boldsymbol{h}_{\triangle} = \begin{bmatrix} a_x & 0 & 0 \\ b_x & b_y & 0 \\ c_x & c_y & c_z \end{bmatrix} = \begin{bmatrix} 60 & 0 & 0 \\ 20 & 70 & 0 \\ 20 & 20 & 80 \end{bmatrix} \tag{S14}$$

The resulting box vector lengths and angles are given in Figure S1A. We then consider 27 identical atoms and place them on a regular $3\times3\times3$ grid, with grid lines parallel to either $\boldsymbol{a}$, $\boldsymbol{b}$, or $\boldsymbol{c}$. This is schematically depicted in Figure S1B. In this geometry, the average distance between atoms is over 20 Å, which is of course much larger than the average atomic separation found in real materials. However, this is intentionally chosen to improve the convergence of the internal stress tensor $\boldsymbol{\sigma}_{\text{int}}$, which has a volume dependence in its denominator (Rogge et al., 2015):

$$\boldsymbol{\sigma}_{\text{int}} = \frac{1}{V}\left[\sum_{i=1}^{N} \frac{\boldsymbol{p}_i \otimes \boldsymbol{p}_i}{m_i} - \boldsymbol{h}_{\triangle}^{T} \frac{\partial U}{\partial \boldsymbol{h}_{\triangle}}\right] \tag{S15}$$

where $\otimes$ denotes the exterior product between two vectors: $(\boldsymbol{u} \otimes \boldsymbol{v})_{ij} = u_i v_j$. Nearest-neighbor interactions are introduced in the system in order to stabilize the crystalline phase shown in Figure S1B. Only two types of interactions are necessary to achieve this: harmonic bonds and harmonic angles. To enforce anisotropic behavior (beyond the strongly triclinic shape of the unit cell), different force constants and rest values are used depending on the direction of the bond or the plane of the angle; these are summarized in Figure S1C. No other interactions were present in the system, i.e. the constituting atoms only interact through the aforementioned short-range covalent interactions. We then proceeded by performing a number of isothermal-isobaric MD simulations, at different pressures. The resulting trajectories were analyzed in terms of the average value of $\boldsymbol{\sigma}_{\text{int}}$ (section 3.1) as well as the volume distributions (section 3.2).

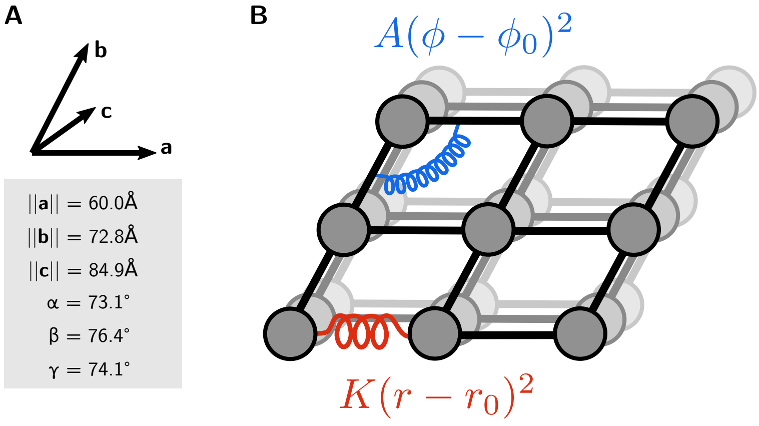

C

	K [kJ/(mol·Å^2)]	r_0 [Å]
along box vector **a**	5	20.0
along box vector **b**	20	24.3
along box vector **c**	1	28.3
	A [kJ/(mol·rad^2)]	ϕ_0 [deg]
in plane **b**-**c**	500	73.1
in plane **a**-**c**	100	76.4
in plane **a**-**b**	200	74.1

Figure S1. **(A)** Description of the (initial) unit cell parameters of the anisotropic crystal. **(B)** Simplified representation of the three-dimensional structure of the crystal. The unit cell contains 27 atoms that are positioned on a regular 3×3×3 grid, with grid lines parallel to the box vectors. **(C)** Parameters for the covalent interactions. Rest values of the interactions are obtained as the actual values in the initial geometry described in (B). For the angles, this implies that both ϕ_0 and $\pi - \phi_0$ are used as rest values, depending on the value of the angle.

3.1 Convergence of the internal stress tensor

Samples that are distributed according to the isothermal-isobaric ensemble satisfy the so-called pressure virial theorem, which states that the average value of $\boldsymbol{\sigma}_{\mathrm{int}}$ equals the externally applied stress $\boldsymbol{\sigma}_{\mathrm{ext}}$ (Tuckerman, 2010):

$$\langle \boldsymbol{\sigma}_{\mathrm{int}} \rangle = \boldsymbol{\sigma}_{\mathrm{ext}} \tag{S16}$$

and for a hydrostatic pressure P, this implies

$$\langle \boldsymbol{\sigma}_{\mathrm{int}} \rangle = P\mathbf{1} \tag{S17}$$

$$= \begin{bmatrix} P & 0 & 0 \\ 0 & P & 0 \\ 0 & 0 & P \end{bmatrix} \tag{S18}$$

We may verify equation S18 by computing the internal stress $\boldsymbol{\sigma}_{\mathrm{int}}$ at regular intervals during a molecular dynamics simulation and monitoring its running average. In doing so, it is useful to decompose the total internal stress $\boldsymbol{\sigma}_{\mathrm{int}}$ into an isotropic and anisotropic contribution:

$$\boldsymbol{\sigma}_{\mathrm{int}} = \frac{\mathrm{tr}\left(\boldsymbol{\sigma}_{\mathrm{int}}\right)}{3}\mathbf{1} + \left(\boldsymbol{\sigma}_{\mathrm{int}} - \frac{\mathrm{tr}\left(\boldsymbol{\sigma}_{\mathrm{int}}\right)}{3}\mathbf{1}\right) \tag{S19}$$

$$= \boldsymbol{\sigma}_{\mathrm{iso}} + \boldsymbol{\sigma}_{\mathrm{a}} \tag{S20}$$

whereby naturally

$$\mathrm{tr}\left(\boldsymbol{\sigma}_{\mathrm{a}}\right) = 0 \tag{S21}$$

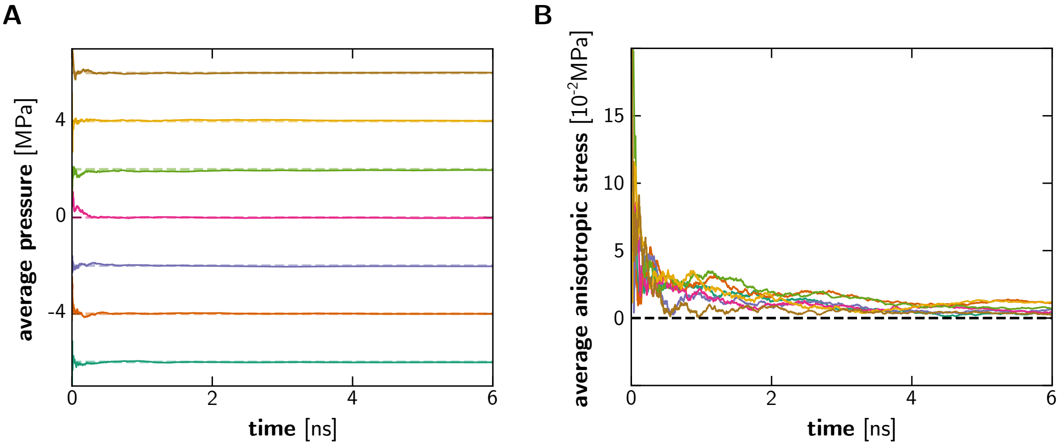

Figure S2. **(A)** Running average of the internal pressure $P_{\mathrm{int}} = \mathrm{tr}\left(\boldsymbol{\sigma}_{\mathrm{iso}}\right)/3$ as a function of simulation time, for seven different pressures (-6 MPa to 6 MPa in steps of 2 MPa). Dashed lines indicate the applied pressure in each of the simulations. **(B)** Root-mean-square of the components of the running average of $\boldsymbol{\sigma}_{\mathrm{a}}$ as a function of simulation time, with colors corresponding to those in (A). All running averages are converging to zero.

Here, it is important to note that the derivative of U with respect to the cell matrix parameters (the second term in equation S15) is not always computed analytically by molecular mechanics engines – such as OpenMM – because it is computationally expensive to evaluate and rather tedious to implement. In those cases, it is necessary to compute the derivative in the virial stress expression (equation S15) numerically, by applying small perturbations in each of the six components and computing the resulting change in potential energy. For numerical reasons, perturbations were performed in the six components of the *symmetric* cell matrix instead of the lower triangular components. Both the running averages of the pressure and the mean squared error of the components of $\boldsymbol{\sigma}_{\text{a}}$ are displayed in Figure S2 for a variety of pressures. We indeed observe that $\boldsymbol{\sigma}_{\text{int}}$ converges to a diagonal matrix, as required by equation S18.

3.2 Ensemble validation

By performing simulations at different temperatures and pressures, we may obtain histograms of observables (e.g. the potential energy or the unit cell volume) for each temperature and pressure. These histograms may then be compared with each other in order to validate whether they are distributed according to the correct Boltzmann distribution (in our case, the (N, P, T) distribution function of Equation S7). This type of ensemble validation was first proposed by Michael R. Shirts (Shirts, 2013), and we refer the reader to his work for a detailed analytical derivation. There, it is shown that the log likelihood ratio of two volume histograms obtained at different pressures satisfies a linear relation that is a function of the temperature and the difference in applied pressure. Here, we report the results of these volume distribution tests in Figure S3. This was obtained using the `physical-validation` Python package[1]. Our results confirm that the proposed pressure control algorithm does indeed generate the correct volume distributions.

$\mathbf{P_0}$ [MPa]	$\mathbf{P_1}$ [MPa]	**analytical slope** [nm^{-3}]	**estimated slope** [nm^{-3}]	**estimated ΔP** [MPa]
-6	-4	-0.4829	-0.4796 +/- 0.0052	-1.99 +/- 0.02
-4	-2	-0.4829	-0.4795 +/- 0.0052	-1.99 +/- 0.02
-2	0	-0.4829	-0.4784 +/- 0.0051	-1.98 +/- 0.02
0	2	-0.4829	-0.4808 +/- 0.0052	-1.99 +/- 0.02
2	4	-0.4829	-0.4805 +/- 0.0050	-1.99 +/- 0.02
4	6	-0.4829	-0.4820 +/- 0.0050	-2.00 +/- 0.02

Figure S3. Results of the ensemble validation procedure (Shirts, 2013). A total of seven simulations were performed at pressures between -6 MPa and 6 MPa, at 300K. Volume distributions were used to determine a maximum likelihood estimate of the pressure difference between two simulations. For each pair of simulations, the estimated pressure difference is in near-perfect agreement with the actual difference in applied pressure.

[1] https://github.com/shirtsgroup/physical_validation/commit/7d3ae2ea71ebaf2cac7b4ab7d922be2c0dfb6639

4 UMBRELLA SAMPLING PROTOCOL

The MIL-53(Al) framework exhibits a large-amplitude structural transition which may be characterized by computing the Helmholtz free energy F as a function of the unit cell volume V using umbrella sampling (see Demuynck et al. (2017)). The interaction energy between particles was modelled using the same force field that was used in the main text. We computed the free energy variation along V using a reference implementation of the MTK barostat using YAFF[2], and a reference implementation of the proposed Monte Carlo pressure control algorithm in YAFF. The calculations were performed on a small $1{\times}2{\times}1$ supercell of the MIL-53(Al) framework. This is done in order to limit the number of particles and avoid the thermodynamic limit (in which case differences between ensembles disappear). Umbrellas were introduced by applying a harmonic restraint to the unit cell volume V:

$$U_H(V) = K(V - V_0)^2 \tag{S22}$$

with $V = \det \boldsymbol{h}$. The center of the harmonic restraint, V_0, was varied between 1480 Å^3 (volume of the cp phase) and 3120 Å^3 (volume of the lp phase), in steps of 20 Å^3. The force constant K was set at 0.0114 kJ/[mol·Å^6], which resulted in a sufficient overlap of the volume distributions between neighboring umbrellas. Resulting trajectories were analyzed using `pymbar` (Shirts and Chodera, 2008) in order to obtain the free energy profile as a function of V. The volume axis shown in Figure 2 in the main text refers to the volume of the $1{\times}1{\times}1$ cell, i.e. half of the $1{\times}2{\times}1$ cell used in the calculations.

[2] available at https://github.com/molmod/yaff/commit/d33861ab81bbe621364a8f58f7b77f65d03206f7

5 OVERVIEW OF PERFORMED SIMULATIONS

Several simulations were performed in order to identify the main transition mechanisms and understand how they depend on the periodicity. Table S2 gives an overview of all simulations. The 37×10×37 system is topologically square, in the sense that the horizontal and vertical direction contain the same number of layers. The 29×10×37 system is slightly elongated in one direction, with 29 layers horizontally and 37 layers vertically. Due to the difference in the number of layers in the horizontal and vertical direction, this model does not allow a single diagonal to transition from the lp to the cp phase because the 29×37 periodicity is not compatible with a single cp layer structure. Because of this, layer-by-layer transitions are prohibited in an artificial manner.

**supercell**	**temperature** [K]	**pressure** [MPa]	**number of atoms**
37×10×37	200	100	1,040,440
37×10×37	200	300	1,040,440
37×10×37	200	500	1,040,440
37×10×37	300	100	1,040,440
37×10×37	300	300	1,040,440
37×10×37	300	500	1,040,440
37×10×37	500	100	1,040,440
37×10×37	500	300	1,040,440
37×10×37	500	500	1,040,440
29×10×37	200	100	815,480
29×10×37	200	300	815,480
29×10×37	200	500	815,480
29×10×37	300	100	815,480
29×10×37	300	300	815,480
29×10×37	300	500	815,480
29×10×37	500	100	815,480
29×10×37	500	300	815,480
29×10×37	500	500	815,480

Table S2. Overview of the performed simulations.

REFERENCES

Demuynck, R., Rogge, S. M. J., Vanduyfhuys, L., Wieme, J., Waroquier, M., and Van Speybroeck, V. (2017). Efficient construction of free energy profiles of breathing metal–organic frameworks using advanced molecular dynamics simulations. *J. Chem. Theory Comp.* 13, 5861–5873. doi:10.1021/acs.jctc.7b01014

Edelman, A. and Rao, N. R. (2005). Random matrix theory. *Acta Numer.* 14, 233–297. doi:10.1017/S0962492904000236

Rogge, S., Vanduyfhuys, L., Ghysels, A., Waroquier, M., Verstraelen, T., Maurin, G., et al. (2015). A comparison of barostats for the mechanical characterization of metal–organic frameworks. *J. Chem. Theory Comput.* 11, 5583–5597. doi:10.1021/acs.jctc.5b00748

Shirts, M. R. (2013). Simple quantitative tests to validate sampling from thermodynamic ensembles. *J. Chem. Theory Comput.* 9, 909–926. doi:10.1021/ct300688p

Shirts, M. R. and Chodera, J. D. (2008). Statistically optimal analysis of samples from multiple equilibrium states. *J. Chem. Phys.* 129, 124105. doi:10.1063/1.2978177

Tuckerman, M. E. (2010). *Statistical mechanics theory and molecular simulation*. Oxford graduate texts (New York: Oxford University Press)

Yot, P. G., Boudene, Z., Macia, J., Granier, D., Vanduyfhuys, L., Verstraelen, T., et al. (2014). Metal–organic frameworks as potential shock absorbers: the case of the highly flexible MIL-53(Al). *Chem. Commun.* 50, 9462–9464. doi:10.1039/C4CC03853C
